# A Computational Model of Attention Control in Multi-Attribute, Context-Dependent Decision Making

**DOI:** 10.3389/fncom.2019.00040

**Published:** 2019-07-10

**Authors:** Kanghoon Jung, Jaeseung Jeong, Jerald D. Kralik

**Affiliations:** ^1^Department of Psychological and Brain Sciences, Dartmouth College, Hanover, NH, United States; ^2^Department of Bio and Brain Engineering, Korea Advanced Institute of Science and Technology (KAIST), Daejeon, South Korea

**Keywords:** decision making, attention, memory, reinforcement learning, cognitive control

## Abstract

Real-life decisions often require a comparison of multi-attribute options with various benefits and costs, and the evaluation of each option depends partly on the others in the choice set (i.e., the choice context). Although reinforcement learning models have successfully described choice behavior, how to account for multi-attribute information when making a context-dependent decision remains unclear. Here we develop a computational model of attention control that includes context effects on multi-attribute decisions, linking a context-dependent choice model with a reinforcement learning model. The overall model suggests that the distinctiveness of attributes guides an individual's preferences among multi-attribute options via an attention-control mechanism that determines whether choices are selectively biased toward the most distinctive attribute (selective attention) or proportionally distributed based on the relative distinctiveness of attributes (divided attention). To test the model, we conducted a behavioral experiment in rhesus monkeys, in which they made simple multi-attribute decisions over three conditions that manipulated the degree of distinctiveness between alternatives: (1) four foods of different size and calorie; (2) four pieces of the same food in different colors; and (3) four identical pieces of food. The model simulation of the choice behavior captured the preference bias (i.e., overall preference structure) and the choice persistence (repeated choices) in the empirical data, providing evidence for the respective influences of attention and memory on preference bias and choice persistence. Our study provides insights into computations underlying multi-attribute decisions, linking attentional control to decision-making processes.

## Introduction

Real-world decisions often require a direct comparison of multiple options, each composed of multiple attributes: for example, selecting among sundry possibilities when grocery shopping by taking cost, personal taste, brand attractiveness, risk with new items, and related information into consideration. For animals more generally, foraging decisions are also based on multiple potential food sources, each with attributes that include extrinsic factors such as location, size, color, and amount, and intrinsic factors such as caloric value and taste. To make decisions in these multi-attribute, multi-option contexts, there are at least two critical barriers. First, such decisions require an efficient process for dealing with the computational load needed to process vast amounts of information. Second, multi-attribute, multi-option decisions require a flexible adaptive-learning process for dealing with various choice circumstances.

Thus, decisions based on larger choice sets require an efficient process for dealing with the computational load to process vast amounts of multi-attribute information. However, such decisions can be quite complex, and humans and non-human animals have evolved cognitive heuristics to simplify the choice set (Kahneman and Frederick, 2005; Chen et al., 2006; Kralik et al., 2012). One key cognitive process used to cope with these computational demands is *selective attention*, which draws a representation of choice options out of the otherwise “blooming, buzzing confusion” of the real world (Corbetta and Shulman, 2002; Wolfe and Horowitz, 2004; Buschman and Miller, 2007; Beck and Kastner, 2009). Attention provides such a process because it plays a critical role in reducing complexity in information processing by focusing on and concentrating relevant information while ignoring other information (Corbetta and Shulman, 2002; Wolfe and Horowitz, 2004; Buschman and Miller, 2007; Beck and Kastner, 2009). Although much is known about attentional processes, it is unclear how attention is dynamically employed to make a decision in larger and various choice contexts (Gottlieb, 2012).

The reinforcement learning framework, which is frequently applied in machine learning and behavioral psychology and neuroscience, has provided a solid background for decision-making models where an agent adaptively learns a behavioral strategy that maximize outcomes (Sutton and Barto, 1998). Although it provides computational, psychological, and neural accounts of conditioned behavior, existing reinforcement learning models lack accounts regarding the mechanism for how multi-attribute options are efficiently evaluated for a decision in various choice contexts.

More specifically, when multiple options are available, choice behavior is partly driven by the context provided by the set of alternatives. The behavioral economics literature has extensively studied context effects and has provided choice models that account for context effects in various choice situations (Chakravarti and Lynch, 1983; Rooderkerk et al., 2011). The context of the choice set can influence choices by affecting how values of options are assessed. For multi-attribute options, each option has different values on different attributes in multi-attribute space. Thus, a decision among multi-attribute options would require comparisons of values of multiple options on various dimensions. But how multi-attribute, multi-option choice problems lead to selective attention allocation and subsequent valuation across the attributes remains unclear.

In addition, memory plays a critical and pervasive role in processing information about options. In multi-attribute decisions under various choice contexts, it is important to efficiently learn the values of multiple options so that improved performance can be achieved. Current reinforcement learning models generally assume that learning is independent of memory already encoded, having a constant learning rate (Sutton and Barto, 1998; Sugrue et al., 2004; Corrado et al., 2005; Lau and Glimcher, 2005; Rutledge et al., 2009). This memory-independent learning could induce inflexibility when values of multiple options need to be learned. In contrast, more efficient learning can be achieved when learning depends on the strength of the memory. But is this more efficient learning actually the case in decision making, at least by animals with higher cognitive capabilities such as primates, and if so, how is the memory-dependent learning realized?

In the current study, we developed a computational model for multi-attribute, multi-option decision making. The model incorporates a selective attention mechanism that determines whether to focus attention on a single attribute or multiple ones based on the relative distinctiveness of the option attributes (such as size, color, or reward value). In addition, the model incorporates memory-dependent learning rates for chosen and unchosen options. We then tested the model on a multi-attribute, multi-option decision-making experiment with rhesus monkeys. We characterized context effects on attribute selection and reward learning in computational terminology, and used the model to capture modulation in preference bias (i.e., the choice distribution) and choice persistence (i.e., the tendency to repeat choices) observed in the empirical data collected with the rhesus monkeys.

## Methods

Animal care and use complied with all current laws and regulations of the United States Department of Agriculture (USDA), and the Institutional Animal Care and Use Committee (IACUC) of Dartmouth College.

### Subjects

Four male rhesus macaques (*Macaca mulatta*) participated in the study. The average age of the monkeys was 8.75 ± 0.48 years (mean ± s.e.m.). They were housed in 32 × 27 × 68 (width × depth × height) inch cages (Allentown Inc., Allentown, NJ, U.S.A.) in a homeroom with automatically regulated temperature, ventilation, humidity, and lighting (14:10 h light:dark cycle, with lights turned on at 06:00 h). The Center for Comparative Medicine and Research (CCMR) at Dartmouth College maintained a full-time animal care and veterinary staff that monitored the monkeys' daily health and well-being. The monkeys were maintained at ~95% of their *ad libitum* weights to ensure sufficient motivation and good health, and their diet consisted of primate chow (no. 5038, PMI Feeds Inc., St. Louis, MO, U.S.A.), supplemented with fresh fruit and vegetables, as well as various treats that included peanuts, cereal, and dried fruits (e.g., raisins, bananas). Environmental enrichment included two or more enrichment items in their home cages at all times, daily playing of radio or videos in the room (the latter via a monitor mounted in view of all individuals), and regular access to a larger enrichment cage (68 × 38 × 72 inches) in an adjacent room.

The monkeys were brought to the testing room in the laboratory in custom-made chairs. The chairs were designed for maximal comfort and safety, so that the monkey's collar slid into a slot that placed the monkey in its preferred natural sitting position, on a perch raised above the floor. The chair loosely restrained the left arm of the monkey while allowing free movement of the right arm. The monkeys were progressively acclimated to the chairs by: (a) initially having them sit near the chairs and eat treats (e.g., raisins, peanuts, fresh fruit, and vegetables) placed on the chairs; and then (b) feeding the monkeys treats when they were first seated in the chairs. After acclimation, the monkeys readily entered the chairs. They exhibited no signs of stress in the chairs and, once seated, displayed natural behaviors, such as normal facial expressions and vocalizations, e.g., food grunts.

For the current study, the chairs were used to: (a) attain precise attention and behavior control across the experimental testing conditions; (b) obtain clear, unbiased choice responses, with tray compartments (described below) at fixed positions relative to the monkey during every trial; and (c) minimize disruption in the test subjects' daily routines, given that they were already acclimated to them from previous experiments. In particular, the location of the food items relative to the monkey was one of the critical option attributes in the experiment, and attention was one of the key processes under study; thus, both needed to be standardized across all trials and conditions. We note that similar chairs have been routinely used in monkey neuroeconomic studies that have successfully replicated multiple behavioral phenomena studied with other paradigms, both in the laboratory and field (e.g., Glimcher et al., 2008; Platt and Ghazanfar, 2010).

### Materials

Each monkey sat across from the experimenter in the chair. An opaque plastic divider separated the experimenter and the monkey, thereby preventing the monkey from seeing the face and upper body of the experimenter. An opening at the bottom of the divider allowed the experimenter to present a transparent plastic food tray to the monkey. The experimenter prepared the tray behind the partition and presented it to the monkey to begin each trial. The experimenter wore a white lab coat, goggles, and a medical mask and gloves and, when presenting, placed his hand in the same position on the back end of the tray (i.e., the end farther away from the monkey). The tray contained four separate compartments, which we labeled according to their positions relative to the experimenter: left (LL); middle left (ML); middle right (MR); and right (RR). During a given trial, food was placed on a circular platform in a compartment so that the monkey could clearly see and easily select its choice of food item. Each compartment was covered by a transparent lid, which the monkey had to lift to gain access to a food item.

Condition 1 used four different types of food: peanut halves (removed from shell) (PN); yellow BioServ® Fruity Gems (FG); BioServ® banana-flavored dustless precision pellets (PL); and rice krispies (KR). The sizes of the four food items were ~0.86, 0.20, 0.12, and 0.50 cm^2^ for PN, FG, PL, and KR, respectively. The calorie amounts per piece were ~2.06, 0.78, 0.15, and 0.08 kcal/piece for PN, FG, PL, and KR, respectively. Condition 2 used FG in four different colors: red, green, orange, and purple; Condition 3 used identical PL for all compartments for three monkeys and PN for the other monkey (Monkey 3) whose motivation was low toward PL. In Condition 3, we excluded the data of the monkey with PN (Monkey 3) for simulation.

### Procedure

For all conditions, four food items were presented to a monkey, with one food item in each tray compartment. To begin the trial, the experimenter slid the tray on the table toward the monkey to a position just out of its reach, and paused for ~3 s for the monkey to observe the items. The tray was then moved toward the monkey to allow it to make a decision by lifting the lid of the compartment and taking the food item contained within. The experimenter then withdrew the tray. After ~3 s to allow the monkey to eat the selected food item, the tray was again slid forward, following the same procedure. Each session consisted of 150 trials, and 10 sessions were conducted, for a total of 1,500 trials. In a few of the sessions, a monkey became satiated before the completion of 150 trials. If the monkey took more than 10 s without choosing a food item, the experimenter noted an omitted trial. If the monkey had three omitted trials in a row, the experimenter stopped the session for the day; extra daily sessions were then carried out to reach the total of 1,500 trials. In Conditions 1 and 2, the arrangement of the food items by compartment was pseudo-randomly determined per session. We recorded all trials in the conditions using MATLAB (MathWorks, MA, U.S.A.) and video recording.

### Data Analysis

#### Empirical Choice Sequence vs. Randomly Shuffled Sequences

For each session, we compared the cumulative run distribution of an empirical choice sequence with that of a randomly shuffled choice sequence to measure the trial-by-trial choice dependency of the empirical data (i.e., a choice history effect), which reflects persistent choice behavior. We calculated the area test statistic that represents the area between two cumulative run distributions in a log-log scale: $A=\int \left|\ln(P_{1}(u)\right)-\ln(P_{2}(u))|\;du$, where *P*_1_ and *P*_2_ are the cumulative distributions of the length of run, *x*, and *u* = ln *x*, similar to a previous study (Malmgren et al., 2008) except we set the minimum value of *P* as 10^−12^ instead of 0 to prevent extreme values from the logarithmic transformation in calculating the area test statistics. First, we obtained 1,000 randomly shuffled choice sequences by randomly shuffling the empirical sequence. Then we computed the average area test statistic between the empirical choice sequence and the randomly shuffled choice sequences. Second, we generated another randomly shuffled choice sequence and identified it as the reference sequence. We then computed the average area test statistic between the reference sequence and the 1,000 randomly shuffled choice sequences. Finally, to determine if the area test statistics for the empirical sequences were significantly different from the area test statistics for the reference sequence, we compared the averaged area test statistic of the empirical sequences with that of the reference sequence across sessions using a paired *t*-test. To determine the overall results for all monkeys, we generated one long sequence by concatenating the sequences of each monkey.

#### Empirical Choice Sequence vs. Sorted Sequences

To examine the degree of dependency across trials in the empirical choice sequence, we also compared the cumulative run distribution of an empirical choice sequence with that of a sorted one. We used the same area test statistic method described above for randomly shuffled sequences, except that instead of using a randomly shuffled sequence, as was done in the reference, the reference sequence was obtained by sorting the empirical choice sequence, e.g., AAABABBCACD becomes AAAAABBBCCD.

#### B- and P-Indices

To quantify the degree of preference bias or persistence in each condition for each individual, we define two indices that reflect the degree of each component behavior: the *B-index* and *P-index*, respectively (Jang et al., 2017). Each index was calculated from the choice sequence of each session. First, the *B-index* is an index of the preference bias, and quantifies the degree of bias toward the option with the highest subjective value vs. an equal sampling of all options. This index captures the idea that the goal-directed process adapts to dynamic valuations by attaining a balance between the exploitation of a high-value option and intermittent exploration of other options in order to detect changed values (Sutton and Barto, 1998; Daw et al., 2006; Frank et al., 2009; Dayan, 2012). We define the B-index based on the following equation: *B-index* $=1-\frac{S_{emp}}{S_{max}}$, where *S*_*emp*_ is the entropy of empirical choice sequences; and *S*_*max*_ is the maximum possible entropy that the choice sequence can have. The entropies *S*_*emp*_ and *S*_*max*_ are computed by the following equation (Shannon, 1948): $S_{emp}=\;-\overset{N}{\underset{i=1}{\sum }}p_{i}log\left(p_{i}\right)$, where *N* is the number of options, and *p*_*i*_ is the choice rate of option *i*; and $S_{max}=\;-log\left(\frac{1}{N}\right)$, where *N* is the number of options. A B-index of 0 would indicate completely exploratory behavior; whereas, an index of 1 would indicate completely exploitative behavior.

The *P-index* is an index of persistence, which quantifies the degree of persistence on an option vs. trial-by-trial independence (Lau and Glimcher, 2005; Rutledge et al., 2009). We define the *P*-index as a ratio with the numerator corresponding to the average of the area test statistics (Malmgren et al., 2008) between cumulative run distributions of the empirical and randomly shuffled choice sequences in a log-log scale, $A_{emp-rand}={\int \left|\ln(P\right)}_{emp}(u)-\ln(P_{rand}(u))|du$, where *P* is the cumulative distribution of the length of run, *x*, and *u* = ln *x*, and the denominator corresponding to the average area statistics between cumulative run distributions of sorted and randomly shuffled choice sequences in a log-log scale, $A_{sorted-rand}=\int |\ln(P_{sorted}(u))-\ln(P_{rand}(u))|du$:

$$\begin{array}{r} \text{P-index}=\frac{A_{emp-rand}}{A_{sorted-rand}} \end{array}$$

A *P*-index of 0 would indicate completely past-independent behavior; whereas, an index of 1 would indicate completely perseverative and past-dependent behavior. Note that since goal-directed behavior is, by definition, based on expected outcome, it should not be solely dependent on previous choices. Thus, the *P*-index should be a relatively pure measure of the persisting component of behavior.

#### Model

The choice model presented here extends a reinforcement learning model using Q-learning that updates an action value for each option based on its prediction error: the difference between the experienced outcome and the action value (Watkins and Dayan, 1992; Sutton and Barto, 1998; Dayan and Abbott, 2001; Daw et al., 2006; Li and Daw, 2011). Similar to previous models (Erev and Roth, 1998; Li and Daw, 2011; Prévost et al., 2011; Jung et al., 2014), the model updates a chosen option based on its reward outcome, and decays unchosen options simultaneously presented in a given context. In addition, the model updates action values of options across all attributes. Thus, at each trial *t*, the action value for the chosen option *c* and for the unchosen option *u* on attribute *k* are updated according to:

$$\begin{array}{r} Q_{k,\;c}\left(\;t+1\right)=\;Q_{k,\;c}\left(\;t\right)+\alpha_{\text{c}}\delta_{c}\left(t\right)\\ Q_{k,\;u}\left(\;t+1\right)=\;Q_{k,\;u}\left(\;t\right)+\alpha_{\text{u}}\delta_{u}\left(t\right) \end{array}$$

where α_c_ and α_u_ are learning rates for chosen and unchosen options and δ_*c*_(*t*) and δ_*u*_(*t*) are the reward prediction errors at given trial *t* for the chosen and unchosen options, respectively.

To capture subjective responses in learning the unchosen option, we assume that the learning rate of the unchosen option is proportional to the action value of the unchosen option in our model: $\alpha_{\text{u}}=\;\alpha_{\text{c}}{Q_{k,\;u}}^{\mu -1}$, where μis the exponent rate parameter that determines how the learning rate is sensitive to the strength of the action value. We used a power law to encapsulate the full range of possible values for the learning rate and to account for all types of subjective responses in learning the unchosen option (Stevens, 1957). The learning rate as a function of the action value of the unchosen option helps explain how the action value influences the learning rate: the larger the action value, the faster the learning; or the smaller the action value, the slower the learning. The exponent μequals the power to which the action value is raised, allowing for response compression for μ < 1, linear response for μ = 1, or response expansion for μ > 1. That is, for μ < 1, the learning rate of the unchosen option decreases as the action value increases; for μ = 1, the learning rate of the unchosen option remains constant regardless of the action value. For μ > 1, the learning rate of unchosen option increases as the action value increases.

The reward prediction errors, i.e., the difference between the expected and received reward values, for the chosen and unchosen options are as follows:

$$\begin{array}{lll} \delta_{c}\left(t\right) & = & \;r_{k,c}-Q_{k,\;c}\left(t\right)\\ \delta_{u}\left(t\right) & = & 0-Q_{k,\;u}\left(t\right) \end{array}$$

where *r*_*k, c*_ is the reward value of the chosen option on attribute *k*. To capture subjective values of reward on each attribute, we assume that there is a power-law relationship between reward value and attribute value of an option *i* on attribute *k* as follows: $r_{k,i}=N{x_{ki}}^{\gamma }$, where γ is the value sensitivity exponent and *N* is a normalizing constant.

Once the action values for each attribute are calculated, we compute an overall action value for an option *i* at trial *t* by summing the action values of the option on all attributes:

$$\begin{array}{l} Q_{i}\left(t\right)=\underset{k}{\sum }{w_{k}Q}_{k,i}\left(t\right) \end{array}$$

where *w*_*k*_ is the weight on an attribute *k*. For multi-attribute decisions, we determine the weight of the action values on each attribute based on relative distinctiveness among options on each attribute as well as its strength.

**Figure 1:**
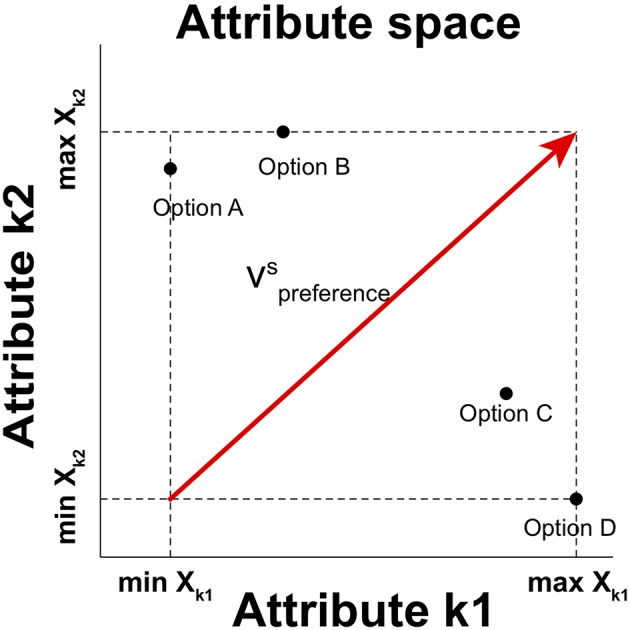
A graphical description of the preference vector for a choice set in two-dimensional attribute space. ${\text{v}}_{\text{preference}}^{\text{S}}$ is the preference vector in the situation of choosing an option in choice set *S* = {Option A, Option B, Option C, Option D}. The horizontal and vertical axes represent the value on distinct Attribute k1 and Attribute k2, respectively. Each option is then represented as a point in this two-dimensional attribute space. The component of the vector on each attribute is determined by the range between the maximum and minimum values of options in the choice set.

We capture the relative distinctiveness by using a preference vector based on valence values of options in choice set *S* (Tversky et al., 1988; Wedel et al., 1998; Roe et al., 2001; Rooderkerk et al., 2011), which connects the initial point that corresponds to the minimum attribute values of all attributes in se t *S* with the terminal point that represents the maximum attribute values of all attributes in set *S* in attribute space (Figure 1). The preference vector in a choice set *S* is

$$\begin{array}{l} v_{preference}^{S}=\left[\left(\underset{i\in S}{\max}x_{1i}-\underset{i\in S}{\min}x_{1i}\right),\;\cdots \;,\left(\underset{i\in S}{\max}x_{ki}-\underset{i\in S}{\min}x_{ki}\right)\right] \end{array}$$

where *x*_*k, i*_ is the normalized attribute value of item *i* on an attribute *k*. Then, the relative distinctiveness of each attribute is determined by the vector component of a unit preference vector on attribute *k*, which is determined by the normalized vector projection of a preference vector on attribute *k* for set *S* in attribute space:

$$\begin{array}{l} u_{k}^{S}=\hat{k}=\frac{\left(\underset{i\in S}{\max}x_{ki}-\underset{i\in S}{\min}x_{ki}\right)}{∥v_{preference}^{S}∥} \end{array}$$

The relative distinctiveness of each attribute depends on the maximum difference between attribute values and the magnitude of the preference vector in a given choice context.

Here, two attentional processes are modeled for attribute selection: *selective attention* and *divided attention* (Corbetta et al., 1991). First, the selective attention process is involved in focusing on a single attribute to the exclusion of other attributes when a single attribute is dominant over other attributes. By using this process, the single dominant attribute is taken into consideration in a winner-take-all manner. Second, the divided attention process is employed in distributing attention to competing attributes simultaneously when no attribute is dominant. These two attentional processes for multi-attribute decisions determine the relative weight of each attribute. A threshold gate is applied to determine which process is used to draw attention on the basis of the relative distinctiveness. If the angle between the preference vector and an attribute axis is below the threshold angle in attribute space, the selective attentional process is employed so that the most distinctive attribute wins and other attributes are disregarded (McCulloch and Pitts, 1943). If the angles θ between the preference vector and all attributes are above the threshold angle Θ_threshold_, the divided attentional process is employed so that attention is distributed according to the magnitude of the projection of the preference vector on each attribute. The angle θ_*k*_ between the preference vector and an attribute *k* is:

$$\begin{array}{r} \theta_{k}=\text{co}{\text{s}}^{-1}\left(\frac{v_{preference}^{S}\cdot \hat{k}}{∥v_{preference}^{S}∥∥\hat{k}∥}\right) \end{array}$$

The output of the threshold gate *a*_*k*_ is:

$$\{\begin{array}{cc} a_{k}=1\;and\;a_{j}=0\text{ for }j\neq k, & \text{if }\theta_{k}<\Theta_{\text{threshold}}\\ \;a_{k}=u_{k}^{S},\; & \text{if }\theta_{k}\geq \Theta_{\text{threshold}} \end{array}$$

**Figure 2:**
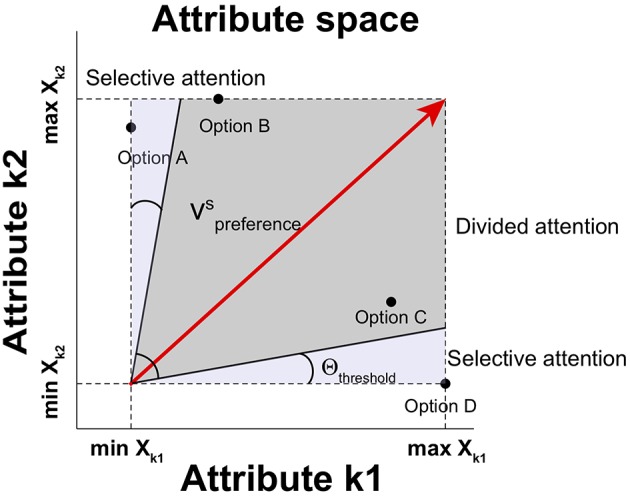
Selective and divided attentional control in attribute space. Depending upon the angle between the preference vector ${\text{v}}_{\text{preference}}^{\text{S}}$ and an attribute in attribute space, different types of attention are employed. When the angle between the preference vector ${\text{v}}_{\text{preference}}^{\text{S}}$ and any attribute falls into Θ_*threshold*_, the attribute is selectively attended. Otherwise, available attributes can be attended simultaneously (producing divided attention).

The relative weight on attribute *k* then is *w*_*k*_ = *a*_*k*_ for the equation of *Q*_*i*_(*t*). An illustration for the two attention processes is shown in Figure 2.

Finally, for action selection, using overall action values of choice options in set *S*, we assume that the probability to choose an option *i* at trial *t*, *P*_*i*_(*t*), is determined according to a softmax choice function (Sutton and Barto, 1998):

$$\begin{array}{r} P_{i}\left(t\right)=\;\frac{exp\left(\beta \sum_{k}{w_{k}Q}_{k,i}\left(t\right)\right)}{\sum_{i=1}^{n}exp\left(\beta \sum_{k}{w_{k}Q}_{k,i}\left(t\right)\right)}=\frac{exp\left(\beta Q_{i}\left(t\right)\right)}{\sum_{i=1}^{n}exp\left(\beta Q_{i}\left(t\right)\right)} \end{array}$$

where β is a softmax inverse temperature parameter, which represents the degree to which choices are biased toward the highest-valued option, and *n* is the number of choice options in set S. Note that, together, different learning rates for the chosen and unchosen options and the relative weights for the option attributes are key components of our model that capture two key features of sequential dynamics in multi-attribute decisions: choice persistence (i.e., repeated choices) and preference bias (i.e., choice distribution), respectively. For estimation of parameters, we calculated the negative log-likelihood of the individual's choice sequence generated by the softmax choice function and used a fmincon function in Matlab to find a constrained minimum of the negative log-likelihood.

## Results

### Behavioral Experiment With Rhesus Monkeys

To better understand the influence of attention and memory on decision-making dynamics, we examined free-choice sequences of multi-option, multi-attribute decisions in rhesus monkeys as they compared different food items in view. Rhesus monkeys are a representative catarrhine primate (comprising Old World monkeys, apes, and humans) whose findings contribute to our understanding of the evolutionary origins of decision-making processes; they are also well-established as a non-human primate model of human decision making (Glimcher et al., 2008; Platt and Ghazanfar, 2010; Xu et al., 2011; Kralik et al., 2012; Jung and Kralik, 2013; Knight et al., 2013). In particular, we addressed the question of how selective attention and memory relate to the two key components of sequential decision making: preference bias, i.e., the overall choice distribution, and choice persistence, i.e., the tendency to repeat choices.

Because selective attention focuses on one or more stimulus attributes at the expense of others, evidence for its effect includes the reduced effect of an attribute on behavior due to the presence of other attributes (Itti and Koch, 2001; Corbetta and Shulman, 2002; Carrasco, 2011). In the extreme, the effect of an option attribute can be completely blocked by others, with a potentially strong effect uncovered once the other attributes are removed (Rescorla and Wagner, 1972; Kahneman et al., 1982; Corbetta and Shulman, 2002; Reynolds and Desimone, 2003; Wolfe and Horowitz, 2004; Slovic et al., 2007; Beck and Kastner, 2009; Hsee and Zhang, 2010; Kahneman, 2011; Kralik et al., 2012). Therefore, to test for the influence of selective attention, we manipulated the relative distinctiveness of option attributes across three conditions, and examined whether there was competition among the attributes in their effects on preference bias and persistence.

**Figure 3:**
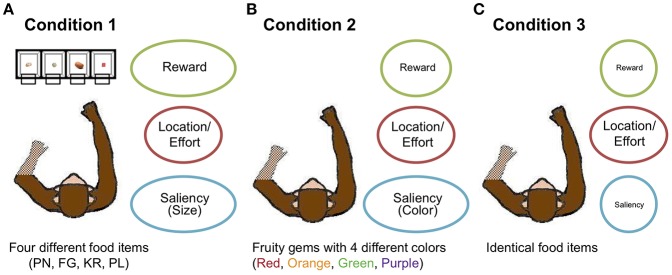
Overview of experimental conditions. **(A)** In Condition 1, the monkeys chose among four different food items (KR, RL, PN, and FG). **(B)** In Condition 2, they chose among four differently colored food items (Orange, Purple, Red, and Green). **(C)** In Condition 3, the monkeys chose among four identical food items. Three attributes—reward (calorie or taste in Condition 1 and possible individual color preference in Condition 2), location/effort, and visual saliency (size in Condition 1 and color in Condition 2)—are chiefly considered. The degree of eccentricity of the ellipses for the three attributes represents the distinctiveness of options with respect to the attributes.

To provide a greater range of attribute distinctiveness, as well as to move toward greater ecological validity in the laboratory by providing multiple options, four monkeys chose among four multi-attribute options (as opposed to the conventional two options) in 1,500 trials in three conditions. In Condition 1, the monkeys chose among four different food items: peanut halves (removed from shell) (PN); yellow BioServ® Fruity Gems (FG); BioServ® banana-flavored dustless precision pellets (PL); and rice krispies (KR) (Figure 3A). The food items differed in both an external perceptual attribute (size) and internal affective ones (caloric amount, taste). Choice could also be affected independently by option location, with items displayed in four transparent containers: left of center (ML), right of center (MR), farther left (LL), and farther right (RR). The locations of the items were counterbalanced across sessions consisting of 150 trials. Thus, although the specific food items changed positions in each session, the effort to reach for items could have led to preferences for the center locations, for example. We then decreased stimulus attribute distinctiveness in two additional conditions. In Condition 2, the monkeys chose among four identical food items that differed in color (and potentially taste): red, green, orange, and purple (Figure 3B). Thus, the size and calorie attributes were eliminated, leaving one highly distinctive external perceptual attribute (color), one slightly distinctive internal attribute (taste), and option location (again, the specific items were pseudo-randomly assigned locations each session, so option location would affect choices independently of the specific items). In Condition 3, they chose among four identical food items, leaving only the location attribute distinctive (Figure 3C): PL for three monkeys and PN for the other monkey (Monkey 3) whose motivation was low toward PL (See Methods for further experimental specifics).

These manipulations were designed to (a) determine whether preference bias and choice persistence were differently affected by external and internal factors, and (b) whether selective attention and memory-dependent learning influenced these two components of sequential choice behavior. Regarding experimental question (a), because preference bias appears to be based on an affective valuation and decision-making process, we hypothesized that it would be most affected by internal factors such as calorie, and would thus decrease across the conditions (with the decrease in internal attribute distinctiveness). Because persistence reflects choice history, i.e., what was selected previously, regardless of the actual item being chosen, we suspected that it might be more immune to affective attributes, and more influenced by general saliency or discriminability of the choice options, regardless of attribute type (i.e., whether internal or external). We therefore hypothesized that choice persistence would be relatively constant in Conditions 1 and 2 (with the high distinctiveness in external perceptual attributes of size and color), but to decrease in Condition 3 (with only location distinctiveness) (Figure 3).

With respect to experimental question (b), evidence for selective attention would be competition among the attributes, such that a particular attribute may have little to no effect on bias or persistence in one condition, but a much greater effect in a subsequent condition, once the more dominant attributes were removed. In fact, in the current experiment, we found evidence for the effect of selective attention on preference bias, in which option location (held constant across conditions) had no discernable effect on preference bias in Condition 1, but exhibited an increasingly stronger effect across Conditions 2 and 3. In contrast, there was no evidence for the effect of selective attention on choice persistence. Finally, evidence for the influence of memory-dependent learning would be tested via the extent to which our computational model captured the monkeys' behavior; and our findings suggest that the memory-dependent learning particularly influenced choice persistence as detailed below.

### Overall Description of Choice Behavior

**Figure 4:**
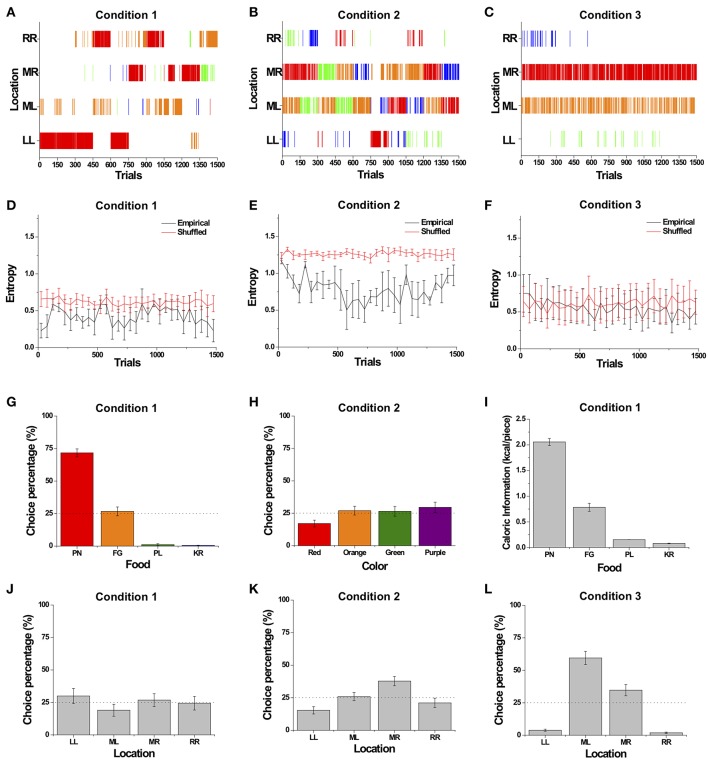
Overall sequential dynamics and preference bias across different choice sets. **(A–C)** The choice patterns of a typical monkey (Monkey 4). **(A)** The dynamic choice pattern in Condition 1 with different food items. Choice in each trial is represented by a tally mark corresponding to the location of the choice made. The tally mark color represents food type: red, orange, green, and blue for peanut halves (PN), fruity gems (FG), pellets (PL), and rice krispies (KR), respectively. **(B)** The dynamic choice pattern in Condition 2 with items of different color. Tally marks are the same colors as the food items. **(C)** The dynamic choice pattern in Condition 3 with identical food items. The choice in each trial is represented by a tally mark corresponding to the location of the choice made. Tally mark color represents location: blue, red, orange, and green for left (LL), middle left (ML), middle right (MR), and right (RR), respectively. **(D–F)** Entropy of choices over trials averaged over all monkeys for Conditions 1 **(D)**, 2 **(E)**, and 3 **(F)** except Monkey 3 for Condition 3. Black and red solid lines represent the entropy of empirical data and randomly shuffled data, respectively. **(G–L)** Preference bias toward a certain attribute averaged over all monkeys. Choice percentage with respect to four food items in Condition 1 **(G)** and with respect to color in Condition 2 **(H)**. **(I)** The caloric information of the four food items. **(J–L)** Choice percentage with respect to location in Conditions 1 **(J)**, 2 **(K)**, and 3 **(L)**. All choice percentages were averaged over all monkeys except Monkey 3 for Condition 3. All error bars are standard error of the mean (s.e.m.).

The choice patterns of a typical monkey (Monkey 4) over the course of Conditions 1, 2, and 3 are shown in Figure 4. Similar to Monkey 4, the decision patterns of all the monkeys generally consisted of a combination of exploitation and exploration among the four food items, as the monkeys made a specific choice for consecutive trials and then occasionally switched to other options. Specifically, in Condition 1 with four different food items, the monkeys exhibited exploration and intermittently showed strong bursty choice behavior, represented as a series of identical choices suddenly occurring, seen as concentrated tally marks of the identical choices (Figure 4A). We observed that monkeys showed a large choice bias to the favorite food item, which provides the highest reward and drives a bursty choice behavior, indicating that their choices were based on the food attribute rather than the distance attribute.

In Condition 2 with four differently colored items, compared with Condition 1, the monkeys showed more exploratory behavior; this, in turn, resulted in a more diverse distribution of choices across colors and locations (Figure 4B). In Condition 3 with four identical items of pellets (PL), the monkeys showed biased choice behavior toward the two middle locations, MR and ML (Figure 4C). However, the monkeys still showed intermittent exploration toward other locations throughout the entire sessions; yet the degree of bursty choice behavior appeared to decrease, compared to those of Conditions 1 and 2. In addition, Monkey 3 with peanut halves (PN) for Condition 3 also showed biased choice behavior toward the MR and ML locations, and intermittent exploration toward other locations throughout the entire sessions, except that the degree of bursty choice behavior appeared to be maintained compared to those of Conditions 1 and 2.

To assess the variation in the degree of the monkeys' biased preference over trials, we calculated *entropy*, a measure of unpredictability in choices, from choice frequencies every 50 trials in all three conditions (Shannon, 1948); a zero value for entropy indicates completely biased preference for choosing a particular option only, whereas a large value represents equal preference over alternatives. In all three conditions, we found that the entropy of choice sequences fluctuated around a particular level throughout the entire sessions (Figures 4D–F). The relatively constant level of entropy over the whole session indicates that the subjects sustained a certain degree of bias in preference throughout the entire session, rather than developing a completely biased preference or completely equal preference.

In addition, we compared the entropy of the empirical choice sequences to a randomly shuffled one, in which any given choice is independent of past choices, in order to examine whether the degree of biased preference or equal preference depended on previous choice history. In Condition 1, we found that the entropies observed in the empirical choice sequences were significantly lower than in randomly shuffled sequences for all four subjects at the individual level, indicating that individual's choices depended on prior choices (paired *t*-test; *p* < 0.001 for Monkeys 1 and 4; *p* < 0.05 for Monkey 2; *p* < 0.01 for Monkey 3) (Figure 4D). In Condition 2, the entropy of the empirical choice patterns was significantly lower than that of the randomly shuffled ones, which again indicates that choices were dependent on past choice history (paired *t*-test; *p* < 0.001 for all four monkeys) (Figure 4E). In Condition 3, the entropy of the choice sequences of the three monkeys with pellets (PL) was maintained at a certain level throughout the whole session; this case, however, did not show a significant deviation from that of randomly shuffled choice sequences for all three subjects with PL (paired *t*-test; *p* > 0.1 for Monkeys 1, 2, and 4), indicating that the empirical choice sequences for the three monkeys with PL appeared to be relatively independent of prior choice history (Figure 4F). However, the entropy of the choice sequences of Monkey 3 with peanut halves (PN) showed a significant deviation from that of randomly shuffled choice sequences (paired *t*-test; *p* < 0.01 for Monkey 3).

Furthermore, we compared the entropy of choice sequences across the three conditions, excluding that of Monkey 3 in Condition 3, whose manipulation (PN vs. PL) differed from the others (see Methods). We found that there was no significant difference in the entropy of the empirical choice sequences across the conditions, indicating that the monkeys showed a similar degree of bias in choices across the conditions [Repeated-measure ANOVA, *F*_(2, 8)_ = 2.815, *p* = 0.119]. However, there was a significant difference in the entropy of the randomly shuffled choice sequences across the conditions [Repeated-measure ANOVA, *F*_(2, 8)_ = 11.875, *p* < 0.01]. The entropy of the randomly shuffled sequences in Condition 2 was significantly higher than those in Conditions 1 and 3 (Tukey *post-hoc* test, *p* < 0.05 between Conditions 1 and 2; *p* < 0.01 between Conditions 2 and 3). This indicates that the degrees of overall bias in the randomly shuffled sequences were different from the global bias in choices across the conditions. In addition, the averaged entropies of the choice sequence for Monkey 3 with PN across conditions were 0.637 for Condition 1, 0.950 for Condition 2, and 0.854 for Condition 3. The averaged entropies of the randomly shuffled choice sequence for Monkey 3 with PN across conditions were 0.720 for Condition 1, 1.30 for Condition 2, and 1.02 for Condition 3.

### Preference Bias

One key component of choice behavior is preference bias toward the options with higher values or ones requiring less effort. To evaluate which attribute of options was most influential in choice behavior, we examined choice percentages with respect to different attributes: food type and location for different food items in Condition 1; color and location for identical food items with different colors in Condition 2; and location for identical food items in Condition 3.

In Condition 1, choice percentages were significantly influenced by the type of food with peanut halves (PN) being consumed the most across subjects, followed by fruity gems (FG) [Two-way ANOVA, *F*_(3)_ = 207.2, *p* < 0.001] (Figure 4G), but not by location [Two-way ANOVA, *F*_(3)_ = 1.66, *p* = 0.178] (Figure 4J). With respect to the factors underlying food type preferences, we calculated the calorie value of the food items presented (Figure 4I). We found that the choice distribution was closely matched to the relative caloric ratios of the food items, i.e., individual calorie value over total calories of all four items (*r* = 0.893, *p* < 0.001) (compare Figures 4G,I). The strong matching between choice percentage of food items and their calorie values indicates a strong bias toward the high-calorie food item. This bias toward the highest caloric food items indicates that the intrinsic value of the food items, particularly caloric value, influenced choice behavior, whereas the absence of spatial bias indicates that the effort needed to reach each location had no significant impact.

In Condition 2, choice percentages for each color were marginally significantly different across all monkeys. In other words, we found that monkeys' preference toward a particular color was marginally significant at the group level [Two-way ANOVA, *F*_(3)_ = 2.59, *p* = 0.055] (Figure 4H). When individual color preference was considered, we found that monkeys showed a strong preference toward an individual's favorite color at the individual level [Two-way ANOVA, *F*_(3)_ = 11.69, *p* < 0.001]. The choice percentages for the four different locations were significantly different [Two-way ANOVA, *F*_(3)_ = 8.37, *p* < 0.001] (Figure 4K). The choice percentage for location MR was significantly higher than LL and RR (Tukey *post-hoc* test, MR > LL, *p* < 0.001; MR > RR, *p* < 0.01) and higher than ML with the margin of statistical significance (MR > ML, *p* = 0.057), indicating that the location of items influenced choice behavior in this condition, unlike in Condition 1 (Figure 4L).

In Condition 3, there was a significant difference in choice percentage across locations [Two-way ANOVA, *F*_(3)_ = 82.2, *p* < 0.001]. ML was chosen significantly more frequently than the other three locations (LL, MR, and RR) (Tukey *post-hoc* test, ML > LL, *p* < 0.001; ML > MR, *p* < 0.001; ML > RR, *p* < 0.001) (Figure 4L). Additionally, the monkey (Monkey 3) with four identical items of peanut halves (PN) in Condition 3 (see Methods) also showed biased choice behavior toward the two middle locations, MR and ML (Choice rate for LL = 0.099; ML = 0.266; MR = 0.585; RR = 0.049; χ^2^(3) = 1,054.0*, p* < 0.001). Comparing Conditions 1–3, the stronger bias toward the two middle locations indicates that the effect of location on choice behavior was insignificant in Condition 1 and then became significant in Conditions 2 and 3, even though the location of options was held constant across all three conditions.

### Choice Persistence

**Figure 5:**
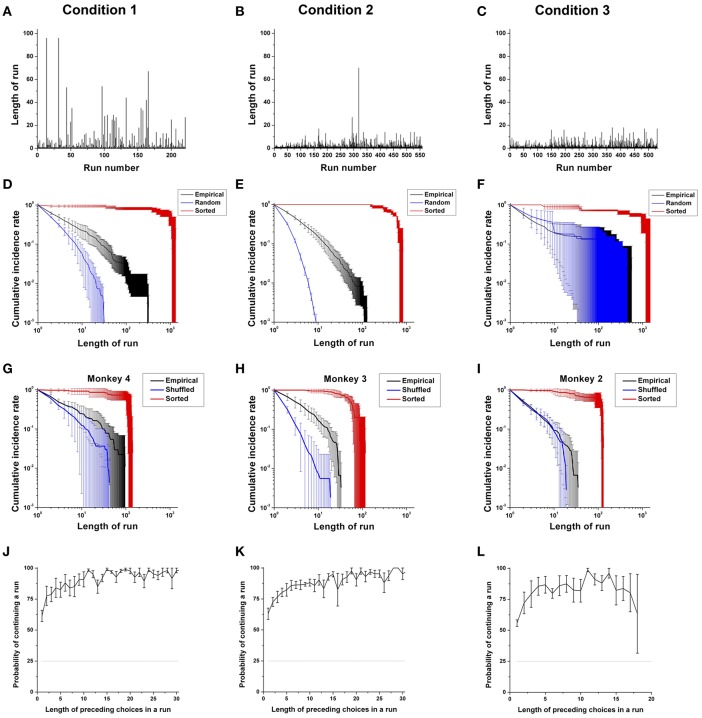
Choice persistence across different choice sets. **(A–C)** A trial-dependent run distribution for a typical monkey (Monkey 4) in Conditions 1 **(A)**, 2 **(B)**, and 3 **(C)**. **(D–F)** The cumulative run distribution of overall choice sequences, as well as those from the sorted and randomly shuffled sequences, averaged across the monkeys for Condition 1 **(D)**, for Condition 2 **(E)**, and for Condition 3 except Monkey 3 **(F)** in a log-log scale. **(G–I)** Example cumulative run distribution of the choice sequence of Monkey 4 for Condition 1 **(G)**, Monkey 3 for Condition 2 **(H)**, and Monkey 2 for Condition 3 **(I)** averaged across sessions in a log-log scale (black line). Red and blue solid lines represent the cumulative distributions of runs obtained from sorted and randomly shuffled choice sequences, respectively. **(J–L)** The probability of continuing a run for the overall sequence and for each rank regarding the most contributing attribute: food in Condition 1 **(J)**; location in Condition 2 **(K)**; and location in Condition 3 **(L)**, as a function of the number of preceding choices in a run for all monkeys except Monkey 3 for Condition 3. The dotted black line represents the chance level of continuing a run (25%).

The second key component of sequential choice behavior is choice persistence: how long an individual continues making the same choices, which we define as a *run*. We observed when monkeys switched to other options and how the length of runs changed throughout the entire session across three conditions (Figures 5A–C). In general, runs consisted of a majority of short runs, as well as a few long runs, for all monkeys. More specifically, in Condition 1, runs consisted of a majority of short runs, as well as a few very long runs (Figure 5A). In Condition 2, while again there were a few long runs, overall the lengths of runs appeared to decrease compared to those of Condition 1 (Figure 5B), reflecting more switching in choice behavior with differently colored items. In Condition 3, the lengths of runs decreased even more compared to those of Conditions 1 and 2 (Figure 5C), and thus the choice behavior with identical items was the least persistent.

On the surface, it is unclear whether increased switching across the conditions is due to a less skewed preference bias or a decrease in choice persistence. That is, bursty sequential dynamics reflect both bias and persistence effects—with a high bias also leading to more and longer runs of identical choices. Thus, the bursty dynamics (as seen in Figures 5A–C) needed to be decomposed into the two components to determine their relative effects across conditions.

To analyze the properties of persistent choice behaviors, we examined the cumulative distribution of the length of runs, which reflects the composition of “switching” and “staying” behavior. Since the distribution exhibited a long and heavy tail at the right end of the x-axis in a linear scale, we rescaled it in a logarithmic scale by using a log-log plot, as shown in Figures 5D–F. To estimate the degree of persistence, we compared the cumulative run distribution of the empirical choice sequences with that of (a) randomly shuffled choice sequences, and (b) sorted choice sequences in all three conditions (see *Data analysis* in Methods). If the cumulative run distribution of an empirical choice sequence does not significantly deviate from that of randomly shuffled choice sequences in which history independency was inherent, this would indicate that choices were made independently of past choice history. On the other hand, if the cumulative run distribution of an empirical choice sequence does not significantly deviate from that of sorted choice sequences, this would indicate that choices were completely dependent on past choice history and were made deterministically.

### Overall Analysis

First, we compared the cumulative run distribution of overall empirical choice sequences with respect to the most contributing attribute (Food in Condition 1; Location in Conditions 2 and 3) with those of the randomly shuffled and sorted ones for the three conditions. In Condition 1, we found that the cumulative run distributions of the overall choice sequences for all four monkeys showed significant deviations from those of the randomly shuffled choice sequences (overall: *p* < 0.001; individuals: *p* < 0.001 for all four monkeys), as well as those of the sorted ones (overall: *p* < 0.001; individuals: *p* < 0.001 for all four monkeys), indicating that the overall long-term choice behaviors of the monkeys were persistent (Figure 5D).

In Condition 2, for all four monkeys, the cumulative run distributions of the overall choice sequences deviated from those of the randomly shuffled sequences, indicating persistence in their overall choices as in Condition 1 (overall: *p* < 0.001; individuals: *p* < 0.001 for all four monkeys); at the same time, there was a high degree of randomness when compared to the sorted sequences (overall: *p* < 0.001; individuals: *p* < 0.001 for all four monkeys) (Figure 5E).

In Condition 3, the cumulative run distributions of the three monkeys with PL in Condition 3 were closest to those of the randomly shuffled sequences (Figure 5F). Generally, the cumulative run distributions significantly deviated from those of the randomly shuffled sequences (overall: *p* < 0.001), indicating the influence of past choice history. The cumulative run distributions also significantly deviated from those of the sorted sequences (overall: *p* < 0.001). However, individually, the cumulative run distribution of the three monkeys with PL did not significantly deviate from those of the randomly shuffled sequences (individuals: *p* = 0.90 for Monkey 1; *p* < 0.001 for Monkeys 2 and 4). Additionally, the cumulative run distribution of Monkey 3 with PN significantly deviated from those of the randomly shuffled sequences (*p* < 0.001 for Monkey 3).

Second, we compared the cumulative run distributions of the empirical choice sequences with those of the randomly shuffled or sorted ones session-by-session. Session-by-session analysis allowed us to test whether the dependence on past choice history was due to a potential artifact from counterbalancing locations across sessions or a prior selection of the most contributing attribute for analysis because the run distribution within a session was not affected by counterbalancing or attributes. In Condition 1, we found that the cumulative run distributions of three of four monkeys showed significant deviations from those of the randomly shuffled choice sequences (overall: *p* < 0.001; individuals: *p* < 0.001 for Monkeys 1; *p* < 0.05 for Monkey 2; *p* = 0.15 for Monkey 3; *p* < 0.01 for Monkey 4), as well as those of the sorted ones across sessions (overall: *p* < 0.001; individuals: *p* = 0.13 for Monkey 1; *p* < 0.05 for Monkey 2; *p* < 0.001 for Monkeys 3 and 4), indicating that the choice behaviors of three monkeys were persistent in each session (Figure 5G). In other words, the choices in each session were significantly influenced by past choice history, thereby revealing some degree of past choice dependency.

In Condition 2, for all four monkeys, the cumulative run distributions deviated from those of the randomly shuffled sequences, demonstrating a choice history effect (overall: *p* < 0.001; individuals: *p* < 0.001 for all four monkeys); at the same time, there was a high degree of randomness when compared to the sorted sequences across sessions (overall: *p* < 0.001; individuals: *p* < 0.001 for all four monkeys) (Figure 5H). Compared with Conditions 1 and 2, the cumulative run distributions of three monkeys with PL in Condition 3 were closest to those of the randomly shuffled sequences (Figure 5I). Generally, the cumulative run distributions did not significantly deviate from those of the randomly shuffled sequences across sessions (overall: *p* = 0.07), indicating the influence of past choice history. However, the cumulative run distribution significantly deviated from those of the sorted sequences across sessions (overall: *p* < 0.001). Individually, the cumulative run distributions of two monkeys did not significantly deviate from those of the randomly shuffled sequences across sessions (individuals: *p* = 0.94 for Monkey 1; *p* = 0.084 for Monkey 2; *p* < 0.001 for Monkey 4). The cumulative run distributions of two monkeys among the three monkeys with PL (see Methods) significantly deviated from those of the sorted sequences across sessions (individuals: *p* = 0.286 for Monkey 1; *p* < 0.001 for Monkeys 2 and 4). In addition, the cumulative run distributions of Monkey 3 with PN significantly deviated from those of the randomly shuffled sequence (*p* < 0.001) as well as the sorted sequence (*p* < 0.001) across sessions.

To further characterize the underlying process for continuing a run, we calculated the conditional probability of continuing a run with regard to the number of preceding choices in the run with respect to the most contributing attribute to preference bias, namely, food in Condition 1; location in Condition 2; and location in Condition 3 (Figures 5J–L). In accordance with the general finding that there was a majority of short runs and a few very long runs, we found that a run was more easily terminated when the length of the preceding choices in a run was short. In contrast, the run was more likely to be continued when the length of the preceding choices in a run was longer.

Specifically, in Condition 1, the probability of continuing a run logarithmically increased as a function of the number of preceding choices in a run and converged to nearly one after a few runs, resulting in long runs (Figure 5J). The increasing probability of continuing a run indicates that the monkeys were more likely to choose what they had repeatedly chosen. As the monkeys repeated their past actions, a *status quo* bias developed in a gradually increasing manner. In Condition 2, similar to Condition 1, we found that the probability of continuing a particular run logarithmically increased with the number of preceding choices in a run (Figure 5K), providing evidence of an increasing tendency of continuing a run as the length of the run increased. In Condition 3, similar to Conditions 1 and 2, we again found that the probability of continuing a run for three monkeys with PL increased with the number of preceding choices in a run. However, we found that the disruption in continuing a run (i.e., a reduction in the probability of continuing a run at a certain run length) occurred for the three monkeys with PL in Condition 3, compared to Conditions 1 and 2 (Figure 5I). This result indicates that the overall tendency of remaining with a previously selected option increased in Condition 3, but that the tendency became progressively unstable as the length of the run increased, compared to Conditions 1 and 2. In addition, we found that Monkey 3 with PN also showed the increasing probability of continuing a run with the number of preceding choices in a run in Condition 3. However, Monkey 3 with PN exhibited a more stable tendency of continuing a run than the other three monkeys with PL without showing an early disruption in continuing a run.

### The Degree of Preference Bias and Persistence

**Figure 6:**
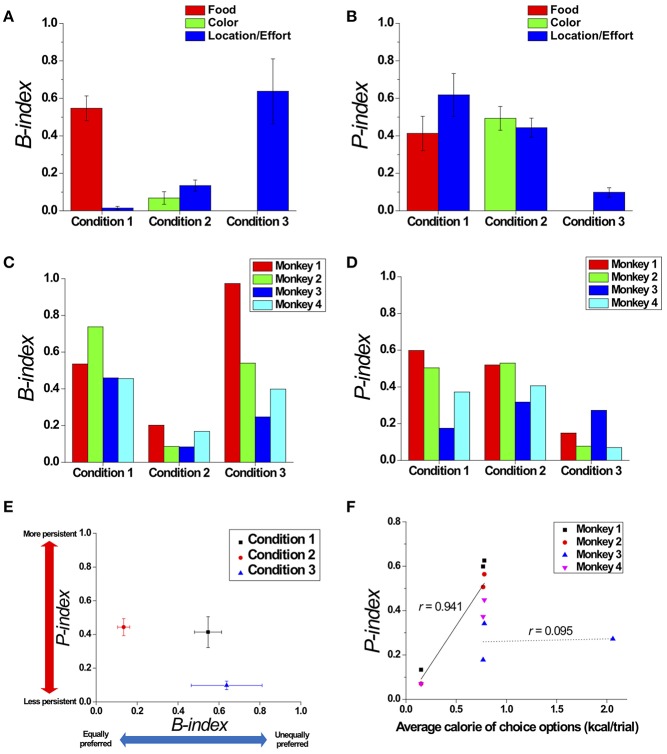
Modulation in preference bias and choice persistence. Comparison of the B-index **(A)** and P-index **(B)** across the three conditions, which reflect preference bias and persistence with respect to certain attributes, respectively. The B-index **(C)** and P-index **(D)** of each individual monkey with respect to the most contributing attribute in each condition: food in Condition 1; and location/effort in Conditions 2 and 3. **(E)** Two dimensional plot of the B-index (x-axis) and P-index (y-axis) with respect to the most contributing attribute, representing the degrees of preference bias and persistence, respectively. All index values were represented as means (± s.e.m.) across all monkeys except Monkey 3 for Condition 3 who received a different manipulation from the others (see Methods). **(F)** The relationship between the average calorie of choice options presented to the monkeys and P-index. The black solid and dotted lines represent linear fits of the data of three monkeys (Monkeys 1, 2, and 4) (*r* = 0.941, *p* < 0.001), and the data of Monkey 3 (*r* = 0.095, *p* = 0.94), respectively.

To quantitatively measure the modulation of preference bias and persistence in choice behavior across the three conditions, we quantified the degrees of preference bias and persistence with respect to a certain attribute by proposing the *B-index* and *P-index*, respectively (see *the B-index* and *P-index* in Methods) (Figures 6A,B). Regarding the preference bias, we found that the B-index of an overall choice sequence with respect to location gradually increased from Conditions 1 to 3 (Figure 6A; Jung et al., 2014). Regarding the choice persistence, we found that the *P-index* of an overall choice sequence with respect to location gradually decreased from Conditions 1 to 3 (Figure 6B).

#### Overall Analysis

To compare the modulation of preference bias and persistence in overall choice behavior across the three conditions, we calculated the B-index and P-index with respect to the most contributing attribute in each condition and plotted them in two-dimensional coordinates (calculated without the data of Monkey 3 in Condition 3, whose manipulation, PN vs. PL, differed from the others—see Methods) (Figure 6E). In both dimensions, we found significant differences in indices across conditions, indicating that the modulation occurred in both preference bias and persistence [One-way ANOVA, *F*_(2, 8)_ = 8.765; *p* < 0.05 for B-index; *F*_(2, 8)_ = 7.156, *p* < 0.05 for P-index]. In particular, we found significant differences in the B-indices between Conditions 1 and 2 (Tukey *post-hoc* test, *p* < 0.05) and between Conditions 2 and 3 (Tukey *post-hoc* test, *p* < 0.05); the B-indices for Conditions 1 and 3 were significantly higher than for Condition 2. In addition, the B-indices of Monkey 3 were 0.460 for Condition 1, 0.084 for Condition 2, and 0.247 for Condition 3. The pattern that the B-index in Conditions 1 and 3 was higher than Condition 2 was also observed at an individual level (Figure 6C).

We also found a significant difference in the P-indices between Conditions 1 and 3 (Tukey *post-hoc* test, *p* < 0.05) and between Conditions 2 and 3 (Tukey *post-hoc* test, *p* < 0.05); the P-indices for Conditions 1 and 2 were significantly higher than for Condition 3. P-indices of Monkey 3 with PN were 0.174 for Condition 1, 0.317 for Condition 2, and 0.272 for Condition 3. The P-index for each individual monkey is shown in Figure 6D. The pattern that the P-index in Conditions 1 and 2 was higher than Condition 3 was also observed at an individual level except for Monkey 3 (Figure 6D).

#### Session Analysis

In addition, we conducted a similar analysis session-by-session in order to test whether the modulation of preference bias and persistence occurred due to the changes of choice sets across conditions and not due to a potential artifact from counterbalancing locations across sessions or a prior selection of the most contributing attribute for the analysis. We calculated the B-index and P-index from the choice sequence of each session, which are uniquely determined within a session irrespective of counterbalancing or attributes. The session analysis consistently showed that there were significant differences in the B-indices of the monkeys across the three conditions (calculated without the data of Monkey 3 in Condition 3 whose manipulation, PN vs. PL, differed from the others—see Methods) [One-way ANOVA, *F*_(3, 107)_ = 36.049, *p* < 0.001]. Specifically, there was no significant difference in the B-index between Conditions 1 and 3 (paired *t*-test, *p* = 0.869). However, the B-index in both Conditions 1 (paired *t*-test, *p* < 0.001) and 3 (paired *t*-test, *p* < 0.001) were significantly higher than in Condition 2. Thus, Conditions 1 and 3 showed similar degrees of preference bias, whereas Condition 2 exhibited the lowest degree. In addition, the averaged B-indices of Monkey 3 across sessions were 0.502 for Condition 1, 0.190 for Condition 2, and 0.292 for Condition 3.

For the session analysis of the P-index, we consistently found significant differences in the P-indices across the three conditions (again, calculated without the data of Monkey 3 in Condition 3, whose manipulation, PN vs. PL, differed from the others—see Methods) [One-way ANOVA, *F*_(2, 107)_ = 3.206, *p* < 0.05]. There was no significant difference in the P-index between Conditions 1 and 2 (paired *t*-test, *p* = 0.241). However, there were also significant differences in the P-indices between Conditions 1 and 3 (paired *t*-test, *p* < 0.01) and between Conditions 2 and 3 (paired *t*-test, *p* < 0.05). The lower P-index in Condition 3 indicates that choice behavior with identical food items was more history-independent and less persistent than in Conditions 1 and 2. In addition, the averaged P-indices of Monkey 3 across sessions were 0.100 for Condition 1, 0.508 for Condition 2, and 0.371 for Condition 3.

We also examined which attributes contribute to choice persistence. We considered all possible attributes for each condition: food rank, food calorie, food size, and location for Condition 1; color rank, color, and location for Condition 2; location rank and location for Condition 3. *Rank* was defined as the order of an individual's overall consumption of each option regarding an attribute, which would reflect the order of an individual's subjective values for the qualitatively different options. We calculated the P-index of each option with respect to these attributes, excluding Monkey 3 in Condition 3. We tested the relationships between rank order and the P-index and found that there were no significant differences in the P-indices for ranks in all three conditions [One-way ANOVA, *F*_(3, 8)_ = 0.723, *p* = 0.566 for Condition 1; *F*_(3, 12)_ = 0.103, *p* = 0.957 for Condition 2; *F*_(3, 6)_ = 0.625, *p* = 0.625 for Condition 3]. In addition, there were no significant correlations between rank order and the P-index in all three conditions (*r* = −0.035, *p* = 0.914 for Condition 1; *r* = 0.066, *p* = 0.809 for Condition 2; *r* = 0.363, *p* = 0.302 for Condition 3). We also found that there were no significant differences in the P-indices for the four locations in all three conditions [One-way ANOVA, *F*_(3, 12)_ = 0.074, *p* = 0.973 for Condition 1; *F*_(3, 12)_ = 1.084, *p* = 0.393 for Condition 2; *F*_(3, 6)_ = 0.647, *p* = 0.613 for Condition 3]. The P-index with respect to location for Monkey 3 in Condition 3 was 0.27.

Further, in Condition 1, we found that there was no significant correlation between calorie of a specific food item and the P-index (*r* = −0.031, *p* = 0.925). We also found that there was no significant correlation between size of the specific food item and the P-index in Condition 1 (*r* = −0.039, *p* = 0.904). In Condition 2, we found that there was no significant difference in P-indices for the four different colors [One-way ANOVA, *F*_(3, 12)_ = 0.164, *p* = 0.919]. However, we found that there was a significant correlation between the average calorie amounts of the presented options and the P-index across the three conditions excluding the data of Monkey 3 in all three conditions (*r* = 0.941, *p* < 0.001) or excluding the data of Monkey 3 in Condition 3 (*r* = 0.802, *p* < 0.01), indicating a positive relationship between the average payoff of available options and choice persistence. For Monkey 3 who experienced peanut halves (PN) in Condition 3 (see Methods), we found that there was no significant relationship between the average calorie amounts of presented options and the P-index (*r* = 0.095, *p* = 0.94) (Figure 6F).

### Modeling Results

Our model incorporates the respective influences of attention and memory on the attribute-selection and reward-learning processes in multi-attribute decisions. To test whether the model can satisfactorily describe these processes, we compared model predictions with empirical data (See Dataset in Methods for details). For the empirical data, as described in “Behavioral Experiment With Rhesus Monkeys”, the four rhesus monkeys freely made choices among four food items in 1,500 trials in three conditions. In Condition 1, four food items of different calorie amounts (FG, PN, KR, and PL) were located in four transparent containers: farther left (LL), left of center (ML), right of center (MR), and farther right (RR). The locations of the items were counterbalanced across sessions consisting of 150 trials. We identified the calorie and the location of items as the main attributes in Condition 1. For the calorie attribute, the caloric value of each presented item was calculated: 0.78, 2.06, 0.08, and 0.15 kcal/piece for FG, PN, KR, and PL, respectively. For the location attribute, we quantified the value of each location based on proximity, which corresponds to a reciprocal value of the actual distance from the monkey to each location. Actual distances were 18, 15, 15, and 18 cm for LL, ML, MR, and RR, respectively; thus the proximities for locations were 1/18, 1/15, 1/15, and 1/18. In Condition 2, four of the same food items in different colors (FG) were located in the four locations (LL, ML, MR, and RR). Similar to Condition 1, the locations of the items were counterbalanced across sessions consisting of 150 trials. In Condition 2, color and location of items were considered the main attributes. Since there are no natural numerical values for colors, we deductively estimated the relative value of each color from its choice rate on the basis of the matching law (Herrnstein, 1961; Jung et al., 2014). The location values were the same as those in Condition 1. In Condition 3, four identical food items (PL) were located in the four locations (LL, ML, MR, and RR). Calorie and location of items were considered the main attributes. The caloric value of PL is 0.15 kcal/piece. The location values are the same as those in Conditions 1 and 2. For Condition 3, we excluded the data of the monkey with peanut halves (PN) (Monkey 3) for simulation, focusing on the three monkeys who received pellets (PL) (see Methods).

**Figure 7:**
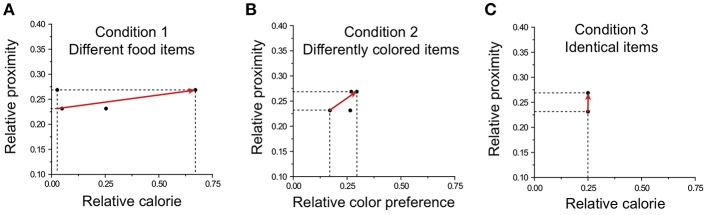
Preference vectors for different choice sets in the three conditions. The preference vector is illustrated with the two most distinguishable attributes for each condition: **(A)** food calorie and location in Condition 1; **(B)** color and location in Condition 2; **(C)** calorie and location in Condition 3.

The preference vector based on the values of each item and location was calculated in each condition. Exemplary preference vectors for three conditions are shown in Figure 7. In Condition 1, the preference vector mostly points toward the calorie attribute (Figure 7A); in Condition 2 the preference vector yields an intermediate angle between the color and location attributes (Figure 7B); in Condition 3, the preference vector points to location (Figure 7C).

For model simulation, we used a reinforcement learning choice model that updates a chosen option based on its reward outcome, and decays unchosen options simultaneously presented in a given context (See Model in Methods). We assigned as free parameters the learning rate constant for chosen options α_*c*_, the exponent for the learning rate μ, the inverse temperature parameter β, and the value sensitivity exponent γ.

Using the preference vector and quantified values of the options on each attribute, the four free parameters of the model were estimated for each individual's data by minimizing the negative log-likelihood of the individual's choice sequence (Table 1; Daw, 2011). The model simulated an individual's multi-attribute decisions in a trial-by-trial manner for each condition. For testing the quality of behavioral fits of choice models, we compared the model with updating action values for both chosen and unchosen options (Q_c+u_) with the model with updating action values for only the chosen option. The Q_c+u_ model produced a lower value of BIC than the Q_c_ model in all three conditions, indicating that the Q_c+u_ model provides a better fit to the behavioral data (Table 2).

**Table 1:** The estimated parameters of the model.

	**α_*c*_**	**μ**	**β**	**γ**
Condition 1	0.79 ± 0.11	2.82 ± 0.14	20.50 ± 1.49	0.09 ± 0.02
Condition 2	0.90 ± 0.04	2.05 ± 0.14	12.37 ± 0.67	0.21 ± 0.08
Condition 3	0.60 ± 0.16	2.51 ± 0.19	20.87 ± 1.41	0.94 ± 0.17

*Values are given as the mean and s.e.m.: α_c_, the learning rate constant for chosen options; μ, the exponent for the learning rate; β, the inverse temperature parameter; γ, the value sensitivity exponent*.

**Table 2:** Quality of behavioral fits of choice models.

	**Model**	**-LL**	**AIC**	**BIC**	**p-r^**2**^**	**Number favoring Q _**c+u**_**
Condition 1	Q _c+u_	574.0 ± 176.9	1,155.8 ± 353.8	1,177.0 ± 354.0	0.72 ± 0.08	4/4 monkeys
	Q _c_	922.2 ± 130.8	1,850.4 ± 261.6	1,866.3 ± 261.6	0.56 ± 0.06	
Condition 2	Q _c+u_	931.8 ± 157.9	1,871.7 ± 315.8	1,893.0 ± 315.8	0.55 ± 0.08	4/4 monkeys
	Q _c_	1,951.8 ± 25.9	3,909.6 ± 51.8	3,925.5 ± 51.8	0.06 ± 0.01	
Condition 3	Q _c+u_	829.6 ± 263.7	1,667.1 ± 527.4	1,688.3 ± 527.4	0.60 ± 0.13	4/4 monkeys
	Q _c_	984.9 ± 335.0	1,975.6 ± 669.9	1,991.6 ± 669.9	0.53 ± 0.16	

*Values are given as the mean and s.e.m. –LL, negative log-likelihood; AIC: Akaike information criterion; BIC: Bayesian information criterion; p-r^2^, pseudo-r^2^ statistic*.

**Figure 8:**
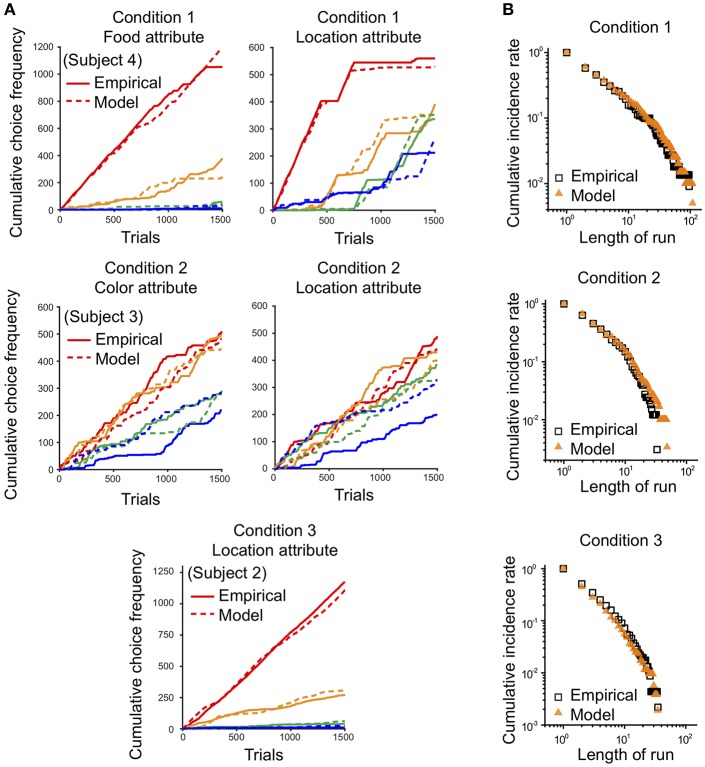
Comparison between empirical choice data and model simulation. Synthetic choice sequences generated from the model were compared with empirical choice sequences. **(A)** Cumulative choice frequencies of the empirical data (solid line) from individuals and simulated data (dashed line) on each attribute in each experimental condition. Red, yellow, green, and blue indicate options on the attribute corresponding to ranks 1, 2, 3, and 4, based on cumulative choice frequency, respectively. **(B)** Cumulative run distributions of the empirical data (square black) and simulated data (orange triangle) shown in **(A)** in each condition in a log-log scale.

The behavior of the model for three conditions is illustrated in Figure 8. First, we compared the cumulative choice graph of simulated data with that of the empirical data with respect to each attribute. The cumulative choice graph of simulated data evolved across trials in a similar way to that of the empirical data on all attributes, indicating that the model captures the dynamic evolution of the multi-attribute choice behavior as well as the overall preference bias among options (Figure 8A). In addition, we compared the cumulative run distribution of the simulated data with that of the empirical data in a log-log scale. The simulations of the model show a close agreement between the cumulative run distributions of the empirical data and the simulated data on all attributes (Figure 8B). This indicates that the model can capture the mechanism that determines how long monkeys continue to choose the same options and when they switch to other alternatives.

**Figure 9:**
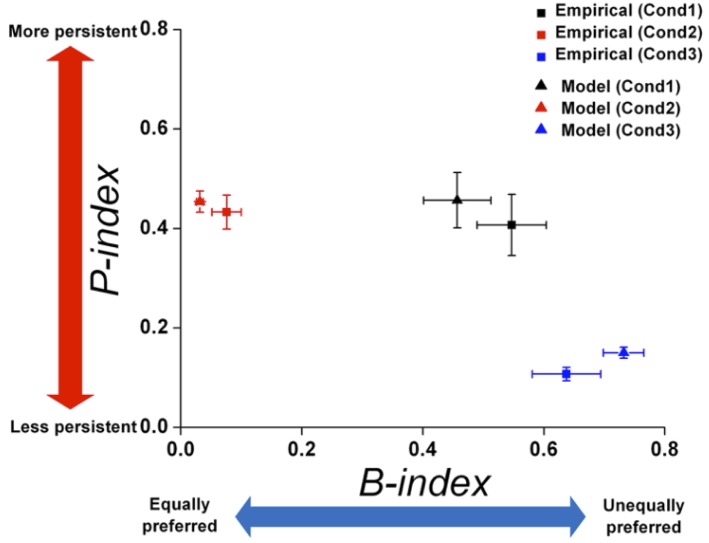
Comparison of *B*-index and *P*-index between empirical data and model prediction. The *B*- and *P*-indices of the empirical data and simulation were compared on each attribute. Two-dimensional plot of the *B*-index (x-axis) and *P*-index (y-axis) with respect to the most contributing attribute: food in Condition 1 and location in Conditions 2 and 3. The squares and triangles represent the results for empirical data and simulation, respectively. Black, red, and blue represent the results for Conditions 1, 2, and 3, respectively. All index values were represented as means (± s.e.m.) across all monkeys except Monkey 3 for Condition 3 who received a different manipulation from the others (see Methods).

Since the empirical data showed that preference bias and choice persistence were modulated across the three conditions, it was necessary to test whether the model could capture these context effects. Therefore, we quantitatively compared the preference bias and choice persistence of the simulation data with those of the empirical data. We calculated the B-index and P-index for the simulation data with respect to each attribute in each condition and compared them with those for the empirical data (see B- and P- indices in Methods for details). The B- and P-indices of the simulation and empirical data are shown in two-dimensional B-P coordinates (Figure 9). In both dimensions, the B- and P- indices predicted from the model closely matched the indices of the empirical data in all three conditions (Paired *t*-test, *p* = 0.246 for B-index, *p* = 0.801 for P-index in Condition 1; *p* = 0.328 for B-index, *p* = 0.648 for P-index in Condition 2; *p* = 0.247 for B-index, *p* = 0.561 for P-index in Condition 3). In addition, the model prediction captured the general patterns in the modulation of preference bias and choice persistence across the three conditions: a high preference bias to specific food items in Condition 1, to specific locations Condition 3, and a robust choice persistence in Conditions 1 and 2.

## Discussion

We examined how selective attention and memory influence the dynamics of multi-attribute decisions—akin to many of the consumer choices in everyday life of humans, and many foraging decisions of non-human animals. Focusing on the influences of attention, here we presented a possible computational account of attention control in multi-attribute decisions. Specifically, we provided the underlying computational mechanisms for how particular attributes are attended and how the values of choice options on multiple attributes are efficiently learned across trials for future decisions. Although prior context effect and reinforcement learning theory have provided accounts for discrete choices in various contexts, and adaptive choice behavior, respectively, the link between the two has not been firmly forged. This missing link is necessary to provide a clearer understanding of how the key cognitive processes lead to multi-attribute decisions. By extending the standard reinforcement-learning model with the addition of attention and memory, this study has constructed a link between context effects and choice behavior based on reinforcement learning.

Our model has two novel components. First, the model includes attentional control for attribute selection. It takes into account the simultaneous evaluation of choice options for multiple attributes in attribute space. The model extends the standard choice model, which has typically been applied to decision making with a single attribute at a time. Applying a preference vector in attribute space (Tversky et al., 1988; Wedel et al., 1998; Rooderkerk et al., 2011), relative contributions for each attribute to choices were calculated. The model suggests that the direction of the preference vector determines whether attention is selectively distributed to the most distinctive attribute or simultaneously distributed to multiple comparable attributes.

An attention mechanism based on a threshold provides an account for how the relative distinctiveness of attributes contributes to a succession of choices. In the behavioral data, the influence of attention on preference bias was evident by the modulation of preference bias on location across conditions. In Condition 1, we found a significant relationship between the preference bias and caloric value of the options, but no relationship with location (or effort). The choice bias toward the favorite food item that provides the highest reward shows that choices were made based on the food attribute rather than the distance attribute. The lack of a relationship with location could be due to the following four factors: (1) the differences in the locations of the food items were not perceptually discernable by the monkeys; (2) the differences were discernable, but too difficult to evaluate and were, therefore, neglected by the valuation system (Hsee, 1996; Tolkamp et al., 1998; Strubbe and Woods, 2004; Zanutto and Staddon, 2007); (3) they were discernable, but given equal values by the decision-making valuation process (e.g., effort was sufficiently similar); or (4) they were discernable, but selectively neglected by an attentional gating mechanism. However, we found a significant effect of location on preference bias in Condition 2, in which the attribute of food items such as calorie was identical, and even more so in Condition 3, in which the attributes of food items such as calorie or color were not distinct features, even though location was held constant across the conditions. Thus, the location differences were discernable, evaluable, and not valuated identically. Therefore, we conclude that the neglect of location in Condition 1, and the increasing effect across conditions revealed the influence of a selective attentional gating mechanism on decision making.

The influence of selective attention on the preference bias is particularly clear when comparing the overall B-indices for Conditions 1 and 3. Even though the choice options were very different, i.e., four qualitatively different (Condition 1) and identical (Condition 3) food items, the B-indices were similar. This is presumably due to the same underlying decision-making process on the attended attributes (e.g., calories in Condition 1 and location in Condition 3): choices were biased to maximize caloric intake in Condition 1 and to minimize the effort to obtain the foods in Condition 3. Thus, it appears that the preference bias was influenced by selective attention.

The influence of attention on choice persistence is not clear. We suggest that attention is involved in spotlighting distinct attributes. Because persistence depends on choice history, i.e., what was selected previously, rather than the preferred attribute, one might suspect that it is more immune to affective benefit and cost influences. As opposed to an increased focusing on the location (or effort) attribute in Condition 3 observed in the preference bias, leading to a preference bias comparable to that in Condition 1, choice persistence decreased in Condition 3. This finding suggests that unlike preference bias, choice persistence appears to be relatively immune to selective attentional effects, and might be more directly and singularly influenced by the general perceptual saliency of the choice options (with high perceptual saliency in external perceptual attributes of size and color in Conditions 1 and 2, and low location saliency in Condition 3). At the same time, another difference between Conditions 1 and 2, on the one hand, and Condition 3, on the other, is average reward outcome across the four options, and there is some evidence for this effect on choice persistence discussed further below. Thus, perceptual saliency and/or average reward outcome may underlie the choice persistence effects, but either way, choice persistence appears to be relatively unaffected by selective attention modulation.

Second, our model suggests that, when a choice is made, learning occurs for both chosen and unchosen options with different learning rates. According to the model, the action values of the chosen and unchosen options are all updated trial by trial based on the prediction error, which signals the difference between the received reward outcome and the action value (Schultz et al., 1997). Further, by assuming memory-dependent learning rates of the value-decay process for unchosen options, a combination of ever-updating action values of chosen and unchosen options generates a preference bias and persistent choices, which correspond to the two key features of sequential dynamics. In particular, our model shows that a memory-dependent learning rate for the value-decay process for unchosen options plays a critical role in generating persistent choice behavior. The standard reinforcement learning model that updates the action value of a chosen option accounts for an underlying control process that guides actions toward a better outcome (Sutton and Barto, 1998). Although some previous studies suggested that the value of the unchosen options may also be updated upon choice (Erev and Roth, 1998; Camerer and Ho, 1999; Hayden et al., 2009; Abe and Lee, 2011; Li and Daw, 2011; Prévost et al., 2011), the process of updating the value of unchosen options has not been given enough attention. Along the lines of our previous study (Jung et al., 2014), the model presented here, emphasizing value-decay for unchosen options as an important learning process, suggests that the decay of action values for the unchosen options results in a greater contrast of action values between the chosen and unchosen options. This increased contrast would generate more momentum in choosing the chosen option again, which in turn leads to persistent choice behavior.

In contrast to current reinforcement learning choice models with constant learning rates, our model suggests memory-dependent learning rates for the value-decay process for unchosen options. Memory-dependent learning rates imply that the value of an option with a higher action value decays more readily when the option is unchosen whereas the value of an option with a low action value hardly changes. In other words, a large decay occurs for more-valued options whereas small decay occurs for less-valued options. Previous studies have suggested that reward-dependent choice behavior can be explained by mechanisms of plasticity (Loewenstein and Seung, 2006; Soltani and Wang, 2006). Thus, the value-decay process might be related to specific mechanisms of synaptic plasticity. A soft-bound synaptic plasticity mechanism by which strong synapses are harder to potentiate than weak ones might be related to memory-dependent learning rates (Fusi and Abbott, 2007; van Rossum et al., 2012). Previous studies have suggested that the soft-bound synaptic plasticity outperforms hard-bound synaptic plasticity in which learning rate is constant (Fusi and Abbott, 2007; van Rossum et al., 2012). Considering behavior as a generic outcome of synaptic plasticity (Loewenstein and Seung, 2006; Soltani and Wang, 2006), the soft-bound synaptic plasticity may explain why the choice model with memory-dependent learning rates may outperform the standard learning model with constant learning rates in complex decision-making situations. Future studies need to examine how soft-bound synaptic plasticity may result in efficient choice behavior.

Having memory-dependent learning rates, it is possible that the value-decay would guide choice behavior based on averaged reward outcomes. Our results showed that a model with these memory-dependent learning rates provides accurate descriptions for choice behavior by capturing choice persistence in the behavior. A previous study suggested that memory-dependent choice behavior is guided by synaptic plasticity based on the covariance between reward and neural activity, and the covariance can be approximated with expected reward value (Loewenstein and Seung, 2006). Thus, in our case, it is possible that choice behavior with different rewards was guided by expected reward value of the choice set, which corresponds to the average reward outcome. This idea is also consistent with a normative perspective on motivational behavioral control based on an average reward reinforcement learning model, which suggests that the average reward rate plays a key role in mediating vigor of responses and implementation of habitual behavior (Niv et al., 2006, 2007). This influence can be reflected in tonic activity levels of dopaminergic neurons (Niv et al., 2006, 2007). Future studies are needed to clarify neurobiological links between choice persistence in memory-dependent adaptive choice behavior and synaptic connectivity.

Although our study has helped clarify the effects of attention and memory on multi-attribute, multi-option decision making, there are limitations to address in the future. One limitation of our study is based on the reward value estimation of qualitatively different affective rewards such as color preference. Our behavioral experiment suggested that individual color preference influenced choice behavior. Compared to other decision variables such as calorie and effort, there is no natural corresponding value for each color. Thus, in the model, we deductively estimated the relative value of colors from their choice frequencies on the basis of the matching law, by which the ratio of behavioral responses matches the ratio of outcome values. However, the accuracy of deductive estimation requires long choice sequences as well as dissociation from the value for other attributes such as location. Although the locations of each color in the behavioral data were counter-balanced across sessions, the value for each color was still confounded with the value for each location within a session. This confound would limit precise estimation for values of qualitatively different rewards. Thus, future experiments designed to eliminate this problem are needed for further validation of the model.

Another limitation is that our model does not take into account other potential perceptual features of the options in the decision-making process. Visual saliency of choice options related to their size, color, texture, and distinctiveness could nudge stimulus-driven bottom-up attention toward a visually salient option. The accumulation of information about available options over time could be considered as costs and possibly influence perceptual decision-making processes when decision time is limited (Drugowitsch et al., 2012). Although our previous study has shown that choice persistence is not directly correlated with specific features of each option including its size, color, calorie, and rank order, it is still possible that the overall distinctiveness among a given choice set influences choice behavior (Jung et al., 2014). Indeed, in our model, each component of a preference vector is determined by the overall distinctiveness of each attribute. Thus, future studies need to clarify the distinct influences of top-down and bottom-up attention on multi-attribute decisions, the process of accumulating information, and the relationships between distinctiveness of attributes and their contributions to choices.

In sum, taking into account influences of attention and memory on decision making, our model provides a plausible computational mechanism for the interplay between attention, memory, and reward in multi-attribute decisions. This study provides insights into the computational mechanisms of cognitive dynamics for effective decisions in complex environments. Our study also points the way for future research to uncover neural mechanisms for how complex multi-attribute information is interactively processed in relation to attention and memory. Such work should lead to the development of therapeutic interventions for poor decision making, resulting from disorders such as attention deficit disorder. Future work based on ours should also continue to help uncover and characterize the mechanisms by which animals negotiate complex, real-world environments—a feat not yet realized in artificial systems.

## Data Availability

All datasets generated for this study are included in the manuscript and/or the supplementary files.

## Ethics Statement

Animal care and use complied with all current laws and regulations of the United States Department of Agriculture (USDA) and the Institutional Animal Care and Use Committee (IACUC) of Dartmouth College.

## Author Contributions

KJ, JJ, and JK conceived of the experimental design, reviewed and revised the manuscript. KJ conducted the experiment, prepared the tables and figures. KJ and JK developed the computational model, analyzed the results, and wrote the main manuscript text.

### Conflict of Interest Statement

The authors declare that the research was conducted in the absence of any commercial or financial relationships that could be construed as a potential conflict of interest.
